# Plasmonic Arrays Resonating at D-Band Communication Frequencies

**DOI:** 10.3390/ma18245679

**Published:** 2025-12-18

**Authors:** Ruxue Wei, Meng Liu, Soren Petersen, Weili Zhang

**Affiliations:** School of Electrical and Computer Engineering, Oklahoma State University, Stillwater, OK 74078, USA; ruxue.wei@okstate.edu (R.W.);

**Keywords:** D-band, terahertz time-domain spectroscopy, extraordinary transmission, plasmonic

## Abstract

We present systematic experimental studies of the impact of subwavelength structural geometries and electromagnetic field polarization on the resonance behavior of metallic metasurfaces at D-band frequencies. The measured influence of the photoconductive receiver antenna design in terahertz time-domain spectroscopy on the frequency-domain spectral features was analyzed. Numerical simulations reveal distinct resonance characteristics in the D-band regime, where extraordinary amplitude transmission is highly dependent on the array dimensions and field polarization orientation. The metasurface enables significant enhancements in surface electric fields and resonance response, attributed to the effective excitation of strong dipolar modes. These results demonstrate the extraordinary transmission capabilities of subwavelength metallic arrays and provide valuable insights for designing compact, low-loss, and tunable terahertz functional components needed in next-generation communications.

## 1. Introduction

Plasmonic subwavelength geometries that enable extraordinary transmission of electromagnetic waves have emerged as unique building blocks of integrated nano- and micro-photonic devices, with a potential for groundbreaking applications in semiconductor nanofabrication, microscopy, display technology, and biochemical sensing [1,2,3,4,5,6,7,8]. When an electromagnetic wave is incident upon the surface of isolated subwavelength structures, resonant transmission may occur due to the excitation of dipolar localized surface plasmons (DLSPs). Under the influence of the electric field, subwavelength structures can be polarized and dipoles are induced as a result, leading to DLSPs [9]. In the low-frequency regime, the resonant excitation of DLSPs in subwavelength structures, characterized by extinction spectroscopy, plays an important role in sixth-generation (6G) communications, surface-enhanced Raman scattering, biosensors, and optoelectronic devices [10,11,12,13,14,15,16].

The D-band frequency range (0.11–0.175 THz) lies within the lower end of the terahertz spectrum and has recently attracted enormous attention due to its unique advantages in short-range ultra-broadband wireless communication, radar target recognition, high-resolution imaging, precision navigation, and electronic countermeasures [17,18,19]. However, effective wave control at these frequencies is challenging due to limited material loss and component scalability. Plasmonic metasurfaces with customized subwavelength geometries provide a promising solution by enabling efficient, tunable resonant transmission in this spectral range.

In this article, we experimentally and numerically investigate the polarization- and size-dependent resonant transmission behavior of plasmonic metasurface arrays designed at D-band frequencies [5,6,7,20,21,22]. Terahertz time-domain spectroscopy (THz-TDS) is employed to characterize the amplitude and polarization responses of subwavelength plasmonic geometries with varying unit aperture widths. CST MICROWAVE STUDIO (CST Studio Suite 2022, Dassault Systèmes) is used to resolve the field distribution and mode excitation mechanisms. We show that the measured transmission enhancement arises from a combination of resonant contributions of DLSP and surface plasmon (SP) interactions and non-resonant backgrounds, such as direct transmission and scattering. Variations in unit aperture width and polarization reveal a clear relationship between structural parameters and resonance strength, thus contributing to a deeper understanding of modal coupling and extraordinary transmission phenomena at the D-band frequencies [6,21].

## 2. Materials and Methods

Plasmonic arrays are fabricated using conventional photolithography and thermal metallization processes onto a 200 nm thick aluminum film with a silicon substrate (0.64 mm thick, n-type resistivity ρ = 20 Ω cm; SEH America, Inc., Vancouver, WA, USA). To minimize the Etalon effect and improve the spectral resolution, a 3 mm thick high-resistivity silicon substrate (n-type, ρ = 10 kΩ cm; Virginia Semiconductor, Inc., Fredericksburg, VA, USA) was attached to the sample with the help of Van der Waals forces to extend the pulse-scanning distance. For a reference in the THz-TDS characterization process, an identical silicon wafer without the aluminum film is joined to the identical 3 mm thick high-resistivity substrate. The size of each sample is 15 × 15 mm^2^, and the unit apertures are fixed at 250 μm length (*l*, along the *y*-axis) with different widths (*w*, along the *x*-axis) ranging from 50 to 350 μm with an interval of 50 μm. The lattice period is constant at 500 μm. The absolute amplitude transmission is defined as

(1) $$\left|\tilde{t}(\omega )\right|=\left|E_{s}(\omega )\right|/\left|E_{r}(\omega )\right|$$

where $E_{s}(\omega )$ and $E_{r}(\omega )$ are the Fourier-transformed frequency-dependent amplitudes of the terahertz pulses and the reference, respectively. The normalized transmission is defined as

(2) $$T_{nor}=max(\left|\tilde{t}\left(\omega \right)\right|)/F_{apt}$$

where $F_{apt}=wl/p^{2}$ for a periodic array with period *p*, unit aperture width *w*, and length *l*. The values $T_{nor}$ > 1 refer to extraordinary transmission. The phase change was obtained through the relation [19]

(3) $$\emptyset (\omega )=\arg\left[\tilde{t}\left(\omega \right)\right]$$

**Figure 1:**
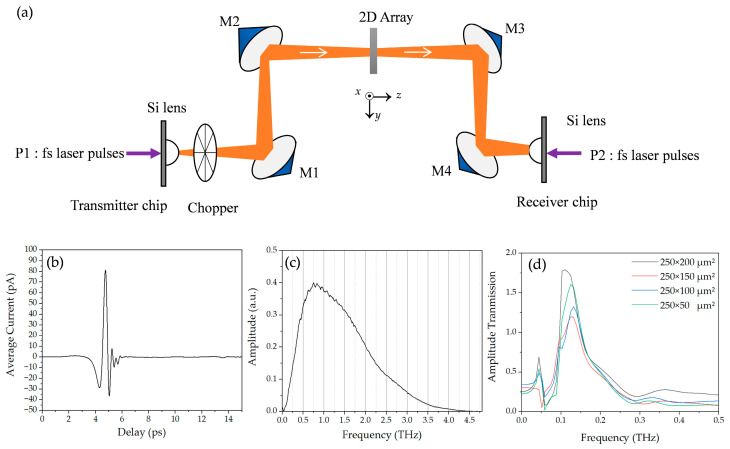
(**a**) Schematic diagram of the terahertz time-domain spectroscopy system (8-F); (**b**) the measured terahertz pulse and (**c**) the corresponding Fourier-transformed spectrum; (**d**) the measured frequency-dependent amplitude transmission of various aperture widths at E//*x* recorded in the THz-TDS 8-F system from (**a**).

**Figure 2:**
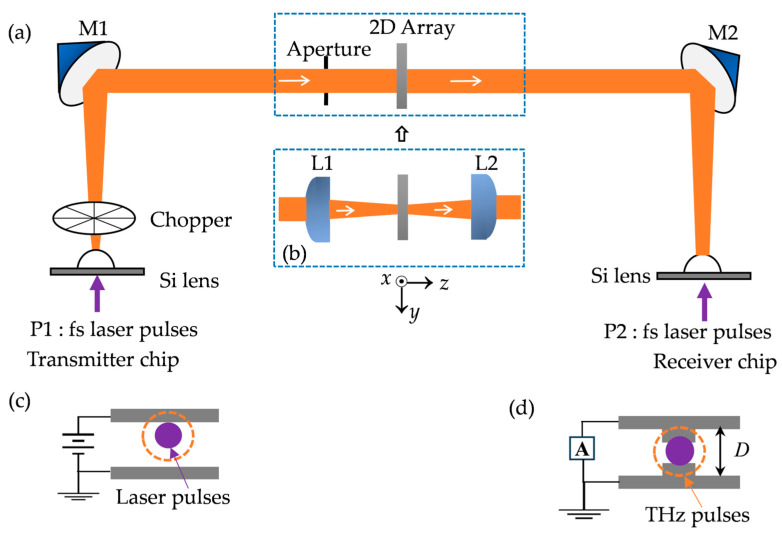
(**a**) Schematic diagram of the terahertz time-domain spectroscopy system; (**b**) addition of a pair of confocal silicon lenses of a 25 mm focal length; (**c**) schematic of the photoconductive transmitter. (**d**) Photoconductive receiver schematic. *D* denotes the dipole length of the receiver antenna.

Broadband and photoconductor-switch-based THz-TDS transmission measurements were analyzed to study the resonance properties of two-dimensional plasmonic arrays [20,23]. As shown in Figure 1a, the eight-focal-length (8-F) THz-TDS system consists of a GaAs-based photoconductive transmission-line transmitter, a sapphire-on-silicon (SOS)-based photoconductive dipole antenna receiver, and four paraboloidal mirrors. The photoconductive transmitter follows the standard dipole-excitation geometry [21], as schematically illustrated in Figure 2c, where the fs pump beam generates carriers in the antenna gap under a DC bias to launch the broadband terahertz pulse. The gate of the photoconductive switch uses a self-mode-locked Ti: sapphire laser capable of generating an 88 MHz repetition rate, 800 nm central wavelength, and 26 fs duration ultrafast optical pulses with an average power of 500 mW under a 4.5 W Nd:YVO_4_ continuous wave 528 nm laser excitation. The average power of the Ti: sapphire laser that excites each side of the photoconductive transmitter and receiver is 10 mW. The terahertz beam from the transmitter is spatially collected by a silicon lens and then collimated into a parallel beam by a paraboloidal mirror M1. To characterize the array samples of small dimensions, the terahertz beam was compressed by an additional pair of F = 50 mm paraboloidal mirrors, M2 and M3, located midway between the two primary paraboloidal mirrors, M1 and M4. The beam was then focused into another silicon lens at the receiver end by M4. The measured terahertz pulse and the corresponding Fourier-transformed spectrum are shown in Figure 1b and Figure 1c, respectively. As shown in Figure 1c, the system has revealed a central frequency of 0.8 THz and a useful bandwidth of 0.1 to 4.5 THz.

Our first set of measurements with the broadband THz-TDS system shown in Figure 1a are illustrated in Figure 1d with the electric field parallel to the *x*-axis (E//*x*) of the aperture, revealing that the sample-to-reference ratio is found to be much greater than 1.0. The apparent higher-than-unity absolute transmission is attributed to the low signal-to-noise ratio around the D-band, where the THz-TDS spectral components are significantly weaker than those near the center frequency.To enhance the D-band frequency components, the low-frequency photoconductive antennas were employed.

**Figure 3:**
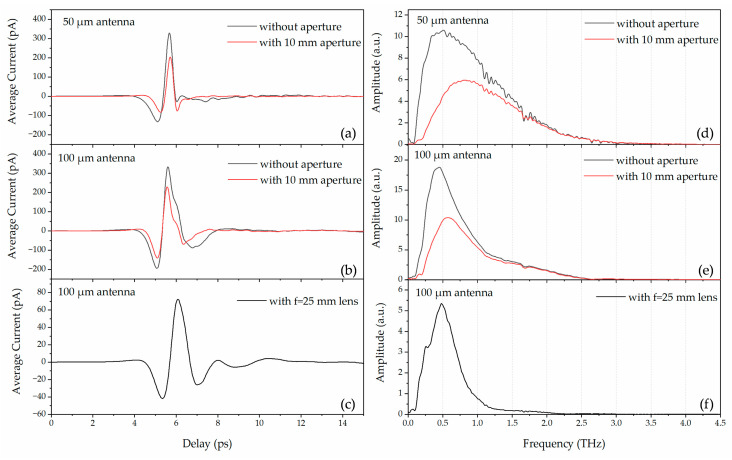
(**a**–**c**) Time-domain terahertz pulses of 50 μm and 100 μm length dipole antenna and (**d**–**f**) the corresponding Fourier-transformed spectra recorded in the 4F THz-TDS system.

As shown in Figure 2a, we utilized a four-focal-length (4-F) THz-TDS system with a parallel terahertz beam between the paraboloidal mirrors M1 and M2. The array sample was fixed to a thin copper plate, centered over a 15 mm diameter aperture in the plate that defined the optical aperture. The system is aligned into a 4F confocal geometry that enables excellent beam coupling between the terahertz transmitter and receiver. At first, the receiver’s photoconductive antenna had a total dipole length of *D* = 50 μm, as shown in Figure 2d. The measured terahertz pulse and the corresponding Fourier-transformed spectrum are shown in Figure 3a and Figure 3d, respectively. As shown in Figure 3d, the system has a central frequency of 0.5 THz (black curve) and an effective bandwidth of 0.1 to 3.5 THz. To minimize boundary reflections and scattering from the sample plate and to ensure that the terahertz beam only illuminates the effective area of the sample, we incorporated a 10 mm diameter aperture in front of the sample plate. However, from the red curve shown in Figure 3a,d, we observed that the signal peak amplitude decreased, especially at D-band. The central frequency was blue-shifted to 0.8 THz, which means the low-frequency signals of interest are at the periphery of the spot and the aperture blocks most of the low-frequency signals.

To further clarify the influence of the photoconductive receiver on the measured spectra, we provide additional details on the antenna geometry, detection mechanism, and calibration method. The receiver in our 4-F THz-TDS setup is a silicon-lens-coupled photoconductive dipole antenna, schematically illustrated in Figure 2d. The device consists of a metal dipole with a total dipole length of *D* = 50 µm (later replaced with a 100 µm dipole for improved low-frequency response) and a sub-micrometer photoconductive gap ~5 µm. When gated by the femtosecond probe pulse, the incident terahertz electric field drives a transient photocurrent, allowing phase-resolved detection of the electric field. The responsivity of a photoconductive antenna is inherently frequency-dependent and is determined by dipole length, carrier lifetime, and the RC time constant. Therefore, the receiver exhibits a pronounced low-frequency roll-off in the 0.1–0.2 THz region. Replacing the 50 µm receiver with a 100 µm dipole increases the effective antenna length and therefore enhances the low-frequency detection bandwidth, consistent with the expected scaling behavior of photoconductive dipoles [24]. This behavior is directly visible in the unnormalized reference spectra shown in Figure 3d–f.

**Figure 4:**
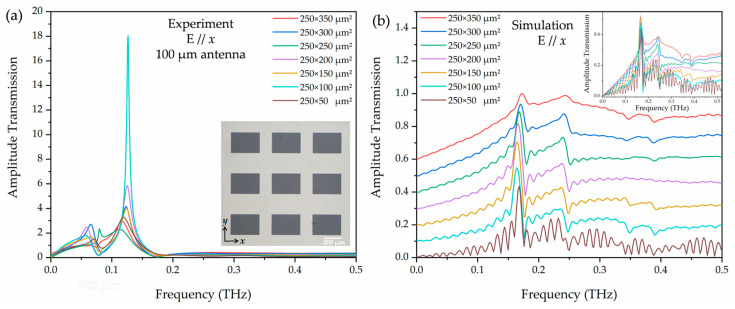
(**a**) The measured frequency-dependent amplitude transmission of various aperture widths at E//*x* recorded in the THz-TDS system with a photoconductive antenna of 100 μm dipole length. Inset: a microscopic image of an array of 250 × 350 μm^2^ and (**b**) the corresponding simulation results. For clarity, the curves are vertically displaced.

**Figure 5:**
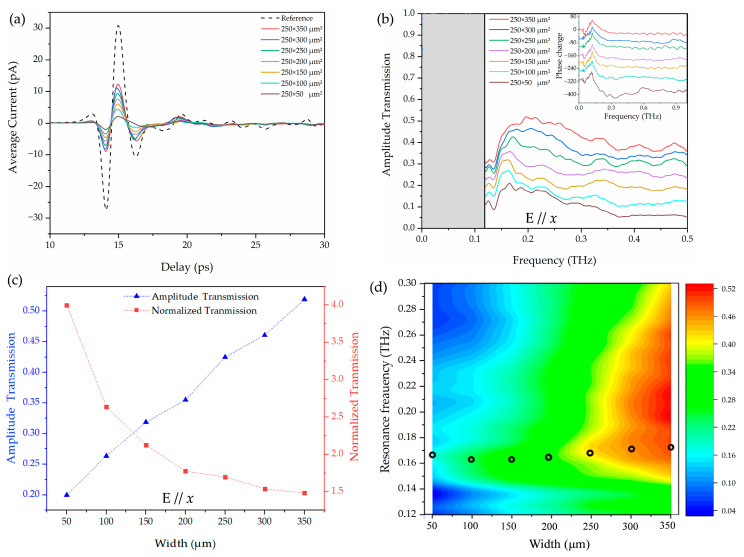
(**a**) The measured transmitted terahertz pulses through the reference and the aperture arrays at E//*x*. (**b**) The measured frequency-dependent amplitude transmissions of various aperture widths at E//*x*. Inset: the corresponding phase change. For clarity, the curves are vertically displaced. (**c**) The measured peak amplitude transmission (blue triangle) and normalized amplitude transmission (red square) as a function of the width at E//*x*. (**d**) The measured resonance frequencies as a function of the width. The circles illustrate the simulated resonance frequencies.

**Figure 6:**
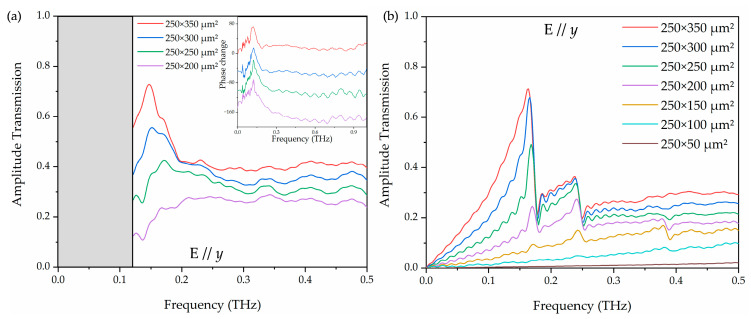
(**a**) The measured and (**b**) simulated frequency-dependent amplitude transmissions of various aperture lengths with a fixed aperture width of 250 μm at E//*y*. The inset of (**a**) shows the corresponding phase change. For clarity, the curves are vertically displaced.

**Figure 7:**
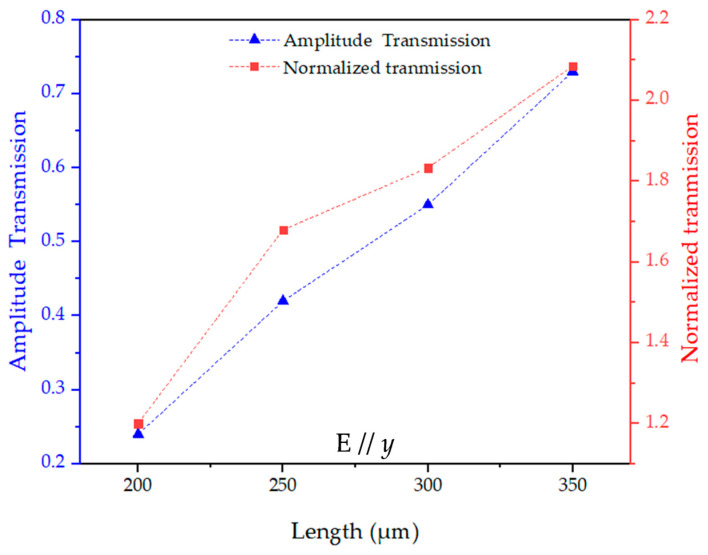
The measured peak amplitude transmission (blue triangle) and normalized transmittance (red square) as a function of the length at E//*y*.

A two-step internal calibration procedure is used. First, a reference waveform is acquired under identical alignment, antenna bias, and pump–probe conditions with a blank substrate identical to the one used for the proposed metasurface. The reference contains the full system response, including the emitter spectrum, receiver responsivity, and optical-path dispersion. Second, the calibrated transmission coefficient is obtained from Equation (1), which removes system-dependent spectral weighting and isolates the sample-induced response. Although the referencing compensates for most instrument effects, the residual low-frequency suppression reflects the intrinsic responsivity limit of the photoconductive receiver rather than sample behavior. This calibration-aware interpretation is applied throughout the analysis presented in Figure 4, Figure 5, Figure 6 and Figure 7.

In order to optimize the system and to enhance the low-frequency components, we replaced the photoconductive antenna with one of a total dipole length of 100 μm. As shown in Figure 3b,e, the black curves represent the time-domain pulse and the corresponding Fourier-transformed spectrum. The central frequency is shifted to 0.4 THz with a full width at half maximum (FWHM) of 0.63 THz. We measured the sample under this condition. The *p*-polarized terahertz electric field is parallel to the *x*-axis, E//*x,* and the unit apertures were fixed at 250 μm in length (along the *y*-axis) with different widths (along the *x*-axis) ranging from 50 to 350 μm with an interval of 50 μm. The inset of Figure 4a shows an image of the 250 × 350 μm^2^ aperture arrays. The lattice constant of the array is 500 μm in both *x* and *y* directions. Similarly, to minimize boundary effects, we incorporated a 10 mm diameter aperture in front of the sample plate, and the experiment results are shown in Figure 4a. As with the 8-F system, near the 0.13 THz frequency, the measured absolute sample-to-reference ratio is much larger than 1, and the results are not repeatable. These differ significantly from the simulation results, as shown in Figure 4b. From Figure 3b,e, we noticed that with aperture (red curves), the central frequency is blue-shifted to 0.6 THz while the aperture of the low-frequency component is greatly reduced, thus leading to an extremely low signal-to-noise ratio that is not able to support accurate measurements of plasmonic transmission properties of the array resonating at D-band frequencies.

According to the Gaussian profile approximation of the terahertz beam [25,26], frequency-independent distribution along the radial direction only occurs in the beam waists. Thus, frequency-dependent distribution is expected at any non-beam-waist location, and the lower the frequency, the larger the beam dimensions. This explains why the low-frequency components below 0.3 THz are significantly cut off by the aperture in front of the sample holder. In order to preserve the low-frequency components and to compress the terahertz beam to a diameter comparable to the size of the sample, a pair of 2-inch-diameter high-resistivity silicon (n-type, ρ = 10 kΩ cm) lenses of 25 mm focal length are placed midway between the two paraboloidal mirrors M1 and M2. As shown in Figure 2a, the original optical setup for the sample (in the dashed box) was replaced with that illustrated in Figure 2b, i.e., the aperture was removed, and a pair of silicon lenses, L1 and L2, were added. As a result, midway between the two lenses, a frequency-independent 7 mm diameter terahertz beam waist is achieved. Figure 3c,f show a measured terahertz pulse and the corresponding Fourier-transformed spectrum. It can be seen that the THz-TDS system has an effective bandwidth of 0.1 to 2.2 THz and the ratio of low-frequency components (0.1–0.3 THz) is noticeably improved when compared with the red curves shown in Figure 3d,e. The overall attenuation in amplitude is due to reflection loss at each of the four surfaces of the silicon lenses. This confocal THz-TDS system with relatively enhanced low-frequency components is employed in the following experimental characterization of our D-band plasmonic array samples.

## 3. Results

Figure 5a illustrates the measured transmitted terahertz pulses through the reference and the D-band plasmonic arrays with various aperture widths ranging from 50 to 350 μm using the THz-TDS system illustrated in Figure 2b. In the THz-TDS characterization, the *p*-polarized terahertz electric field is parallel to the *x*-axis, E//*x*, as shown in the inset of Figure 5a. The lattice constant of the array is 500 μm in both the *x* and *y* directions. Due to the limited low-frequency response of the photoconductive antenna, the measured data below 0.12 THz, shaded in Figure 5b and Figure 6a, are not considered accurate enough. Furthermore, to enable a fair comparison between the simulation and experimental results, we restricted the linewidth analysis to the 0.12–0.5 THz frequency range in order to better match the effective measurement bandwidth of the THz-TDS system.

Figure 5b shows the frequency-dependent amplitude transmissions of the Fourier-transformed terahertz pulses and phase change (inset). It is interesting to note that the measured absolute amplitude peak transmission exhibits a significant increase as the aperture size increases. Figure 5c shows the absolute and normalized peak transmission amplitude as a function of the aperture width acquired from the measurements. When the array length is fixed at 250 μm, the array width has a broad effect on the field enhancement. Several well-defined resonant features were observed and essentially attributed to the resonant excitation of SPs at terahertz frequencies. The absolute (blue scatters) and normalized (red scatters) amplitude transmission approach the maximum at 250 × 350 μm^2^ and 250 × 50 μm^2^, respectively. The sharp resonance near 0.17 THz exhibits 4.0 normalized amplitude transmission efficiency for the width of 50 μm. The observed transmission enhancement properties of the aperture arrays are essentially the result of the excitation of the DLSP resonance of the unit apertures and their coupling to the SPs and direct scattering. Increasing the unit aperture width along the direction of the electric field enhances its DLSP resonance, which in turn produces a resonance enhancement through coupling to the SPs and DLSPs of the apertures.

Figure 5d illustrates measured resonance frequencies as a function of the width of the unit aperture; the circles are the simulated resonance frequencies from Figure 4b. The simulated resonance frequency fluctuates around 0.165 THz, which agrees well with the experimental data. Quantitatively, the resonance frequency differs between the experiment and simulation by less than 0.004–0.005 THz across all widths (<3.2% deviation). The numerical simulation parameters are defined by our THz-TDS system. Additionally, we observed that the peak amplitude increased with increasing width until 200 μm and then decreased with further increasing width to 350 μm in the simulation results (Figure 4b inset), rather than simply increasing with increasing aperture width in the experiment. The experimental peak transmission amplitudes exceed the simulated values by 0.03–0.08 across all aperture widths, corresponding to a relative deviation of 19–33%. This may be due to the ideal periodic and absorbing boundary used in the simulation. In the experiment, the array is finite and scattering still exists at the edges. Therefore, the turning point in the simulation may be the intrinsic behavior of the internal resonance of the structure but is masked by other phenomena in the experiment.

For clarification of the residual differences between the measured and simulated spectra, we note that the experimental beam illuminates only a finite 7 mm × 7 mm = 49 mm^2^ area, whereas the numerical model assumes an infinitely large periodic array. This finite illumination introduces edge effects and non-uniform boundary fields that are absent in the periodic simulation. In addition, the low-frequency part of the THz-TDS spectrum is strongly affected by the responsivity and dynamic range of the photoconductive receiver. Earlier THz-TDS studies have similarly reported that the transmitted low-frequency components of the THz pulse are inherently weak and exhibit a reduced dynamic range, and that reliable measurements below 1 THz often require extended receiver antenna lengths to improve the signal-to-noise ratio (SNR) [24]. This evidence in the literature is fully consistent with our observation that the 0.1–0.2 THz region exhibits the largest residual deviation. Finally, small fabrication-related variations, such as metal thickness, sidewall tapering, and substrate loss, further contribute to the amplitude difference. These combined factors account for the remaining mismatch between the experiment and simulation.

To investigate the polarization effect, the amplitude transmission with terahertz electric field switched to E//*y* was measured and simulated, as shown in Figure 6. Comparing the peak amplitude transmission and normalized transmittance of Figure 5c and Figure 7, owing to the polarization-dependent nature of the rectangular apertures, the arrays exhibit much stronger peak transmission of E//*y* at 250 × 350 μm^2^ (0.73) than that of E//*x* at 250 × 350 μm^2^ (0.52), corresponding to a 40% enhancement. The other aperture sizes also show 18–28% higher transmission under E//*y*, confirming the strong polarization sensitivity of the rectangular hole arrays. The measured highest normalized transmittance for E//*y* was obtained in the array of 250 × 350 μm^2^, having a value of 2.1 at 0.15 THz. In the amplitude transmission shown in Figure 6a, there is no obvious resonance peak when the width is less than 250 μm at E//*y* due to dependence of the resonance intensity on the polarization of the electric field. This behavior is also evidenced by the simulation results shown in Figure 6b.

**Figure 8:**
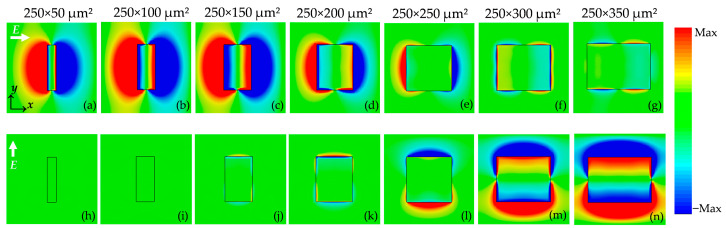
Instantaneous real-part electric field distributions at the resonance frequency for rectangular apertures with widths from 50 μm to 350 μm under (**a**–**g**) *E*//*x* and (**h**–**n**) *E*//*y* excitation.

The plasmonic mechanisms governing the width-dependent transmission in Figure 8 present the instantaneous real-part electric field distributions at 50 μm above the surface for different aperture widths under the two orthogonal polarizations. Under E//*x*, as seen in Figure 8a–g, a clear dipolar pattern with opposite-sign lobes is formed along the long edges of the aperture. This is the characteristic signature of DLSP excited by the electric field. For narrow apertures (50–150 µm), these dipolar lobes are strongly localized at the metal edges, indicating efficient DLSP excitation and a large near-field enhancement per unit aperture area. As the aperture width increases, the dipolar contrast gradually weakens and the field inside the aperture becomes more uniform, reflecting a reduced contribution of DLSP-mediated tunneling relative to the direct geometric opening. This evolution is fully consistent with Figure 5c: although the absolute transmission increases with width, the normalized transmission, which reflects the extraordinary transmission per unit area, decreases because the DLSP contribution becomes diluted in wider apertures.

In contrast, when the incident field is rotated to E//*y*, as seen in Figure 8h–n, the apertures do not support a pronounced dipolar pattern along the short side, and only weak edge-localized fields are observed for widths below 200 µm. The widest apertures (300–350 µm) indicate that a strongly propagating DLSP mode is formed. This polarization-selective DLSP mode directly explains why the E//*y* transmission exhibits width dependence in Figure 7. Overall, the near-field distributions in Figure 8 confirm that the extraordinary transmission observed in our metasurface originates predominantly from the DLSP excitation along the long edges of the apertures.

## 4. Conclusions

We systematically investigated the influence of subwavelength rectangular aperture dimensions and terahertz electric field polarization on the resonant transmission properties of metallic metasurface arrays in the D-band using the low-frequency THz-TDS platform. Both experimental measurements and numerical simulations reveal that the transmission characteristics are strongly dependent on the aperture width and electric field polarization orientation.

For the electric field polarized along the *x*-direction (E//*x*), the amplitude transmission increases with increasing aperture width, with a dominant resonance peak observed near 0.165 THz. Extraordinary transmission, exceeding unity when normalized to the aperture area, was attributed to the combined excitation of DLSP resonances, as well as direct scattering. For *y*-polarized fields (E//*y*), stronger transmission and sharper resonances were observed, especially at a width of 350 μm, confirming the polarization-sensitive nature of the rectangular aperture arrays. Field distribution simulations further verify that DLSP resonance strength diminishes significantly with decreasing aperture width.

These findings provide valuable insights into the resonance mechanisms of subwavelength terahertz metasurfaces. The tunable resonance behavior with transmission enhancement observed in the proposed metasurface structures is promising for terahertz communication applications. The resonance frequency and field confinement can be precisely controlled, leading to the design of frequency-selective surfaces with tailored spectral response [10,27,28].

Practically, the proposed metasurface has applicable relevance for D-band components. First, the tunable resonance near 0.17 THz allows the structure to operate as a compact frequency-selective surface for short-range 6G communication. By adjusting the aperture width, the passband position and transmission strength can be lithographically controlled, enabling functions such as interference suppression, channel preselection, and spectral shaping for high-data-rate transceivers.

Furthermore, the strong polarization anisotropy observed in our arrays makes the device suitable for polarization-sensitive terahertz modules, including compact amplitude modulators, polarizing beam splitters, and polarization-encoded imaging elements. Such components are important in high-resolution THz radars, coded-aperture imaging, and polarization-division-multiplexed wireless communication. These representative use cases demonstrate that the proposed metasurface is not limited to fundamental studies but can be directly integrated into practical D-band systems.

## Data Availability

The original contributions presented in this study are included in the article. Further inquiries can be directed to the corresponding author.
